# Socially Assigned Race and the Health of Racialized Women and Their Infants

**DOI:** 10.1089/heq.2022.0079

**Published:** 2022-11-18

**Authors:** Nadia N. Abuelezam, Adolfo Cuevas, Sandro Galea, Summer Sherburne Hawkins

**Affiliations:** ^1^ William F. Connell School of Nursing, Boston College, Chestnut Hill, Massachusetts, USA.; ^2^ Community Health, Tufts University, Medford, Massachusetts, USA.; ^3^ School of Public Health, Boston University, Boston, Massachusetts, USA.; ^4^ School of Social Work, Boston College, Chestnut Hill, Massachusetts, USA.

**Keywords:** maternal health, racism, infant health

## Abstract

**Introduction:**

While historically most public health research has relied upon self-identified race as a proxy for experiencing racism, a growing literature recognizes that socially assigned race may more closely align with racialized lived experiences that influence health outcomes. We aim to understand how women's health behaviors, health outcomes, and infant health outcomes differ for women socially assigned as nonwhite when compared with women socially assigned as white in Massachusetts.

**Methods:**

Using data from the Massachusetts Pregnancy Risk Assessment Monitoring System (PRAMS) Reactions to Race module, we documented the associations between socially assigned race (white vs. nonwhite) and women's health behaviors (e.g., initiation of prenatal care, breastfeeding), women's health outcomes (e.g., gestational diabetes, depression before pregnancy), and infant health outcomes (e.g., preterm birth, low birth weight [LBW]). Multivariable models adjusted for age, marital status, education level, nativity, receipt of Special Supplemental Nutrition Program for Women, Infants, and Children (WIC) assistance during pregnancy, infant sex, plurality, and gestational age. Additional models adjusted for treatment by race, how often one thinks about race, and nativity.

**Results:**

Women socially assigned as nonwhite had higher odds of breastfeeding (adjusted odds ratio [AOR]: 1.86, 95% confidence interval [CI]: 1.54 to 2.25), lower odds of consuming alcohol (AOR: 0.27, 95% CI: 0.24 to 0.31), and lower odds of smoking (AOR: 0.30, 95% CI: 0.24 to 0.38) compared with those socially assigned as white. However, women socially assigned as nonwhite had higher odds of reporting gestational diabetes (AOR: 1.97, 95% CI: 1.49 to 2.61). Mothers socially assigned as nonwhite also had higher odds of giving birth to an LBW (AOR: 1.66, 95% CI: 1.29 to 2.14) and small-for-gestational age (AOR: 1.46, 95% CI: 1.19 to 1.80) infant compared with women socially assigned as white.

**Discussion:**

In comparison with women socially assigned as white, we observed poorer health outcomes for women who were socially assigned nonwhite despite engaging in more beneficial pregnancy-related health behaviors. Socially assigned race can provide an important context for women's experiences that can influence their health and the health of their infants.

## Introduction

Black women in the United States experience higher maternal morbidity and mortality rate than their white peers as do their infants. For example, black women are more likely to be diagnosed with severe maternal morbidity or receive life-saving procedures (e.g., renal failure, embolism, eclampsia)^1,2^ and have a 2.4 times higher risk of maternal mortality rate compared with white women.^3^ Furthermore, Latina woman in the United States experience disparities in maternal and infant health outcomes that are stratified by racial grouping.^4–6^ Socioeconomic status (e.g., income and education) has long been shown to help explain racial disparities. However, racial disparities persist even after adjusting for socioeconomic status,^7–9^ suggesting that forces on the spectrum from individual and institutional discrimination to structural racism are important social determinants of maternal health over and above the role of socioeconomic status.^10^

Race is a multidimensional and sociopolitical construct that is part of a larger organized system to allocate goods and resources to certain racial groups.^11^ While historically most public health research has relied upon self-identified race as a proxy for racism, a growing literature recognizes that socially assigned race (also known as “socially ascribed” or “what others think you are” race) may more closely align with racialized lived experiences that influence health outcomes.^12,13^ Discrepancy is common among racial and ethnic minority group members between their self-identified race and socially assigned race.^14,15^ This discrepancy can act as a stressor and induce compromising maternal health behaviors (e.g., smoking), as we found in our previous work with women in this sample.^15^

Thus, socially assigned race can capture the structural and interpersonal influences that affect minoritized individuals in a racialized society.^12,16^ In other words, members of the same racial/ethnic group may have different racialized lived experiences based on how society perceives them.^16^

Socially assigned race has been found to predict discrimination experiences and health care utilization.^13^ Other studies have found that individuals socially assigned as white experience health advantages even if they self-identify as nonwhite.^16^ Emerging research suggests that nativity status may play a role in the association between socially assigned race and health.^17^ For example, being socially assigned white was positively associated with health among Mexican-born Latinx individuals but not among US-born Latinx individuals in a national survey.^17^ Maternal and infant health is among the health indicators that are most sensitive to social conditions. The disproportionate burden of poor maternal and infant health disparities experienced by black and Hispanic women and infants reflects long-standing societal inequities. Socially assigned race, alongside experiencing racism and discrimination as a result of that assigned race, can provide additional evidence for how racialization influences maternal health.

We aim to understand how women's health behaviors, health outcomes, and infant health outcomes differ for women socially assigned as nonwhite when compared with women socially assigned as white in Massachusetts. We analyze these differences in the context of other racial experience variables, including how often a woman thinks about race, and stress related to treatment by race and nativity (hereafter, referred to as background stress). We hypothesized that women who were socially assigned as nonwhite would be less likely to have early prenatal care, use alcohol or smoke before pregnancy, but more likely to breastfeed; they would be more likely to have gestational diabetes, but less likely to report depression before or after pregnancy; and their infants would have poorer birth outcomes than infants whose mothers were socially assigned as white.

## Methods

The data for this study originate from the Massachusetts Pregnancy Risk Assessment Monitoring System (PRAMS) data. PRAMS survey data are linked to birth certificate data and include questions to understand behaviors and experiences before, during, and after birth. Between 2012 and 2015, the Reactions to Race module was incorporated into the PRAMS survey, which asked about racial lived experiences, perceptions about race, and thoughts about race.

### Socially assigned race

Women were categorized as being socially assigned as white or nonwhite as the main exposure of interest. One of the primary questions on the Reactions to Race module asked “How do other people usually classify you in this country? That is how other people usually classify you in this country, which might be different from how you classify yourself,” with response options as follows: white, black or African American, Hispanic or Latina, Asian or Pacific Islander, American Indian, or other. If a woman selected white, she was considered to have a socially assigned race as white. If a woman selected any other category, she was categorized as having a nonwhite social ascription. Women were only allowed to select one response category for socially assigned race.

### Outcome measures

The main outcomes of interest were categorized into women's health behaviors, women's health outcomes, and infant health outcomes, and have been described in prior work (all dichotomous).^15^ Women's health behaviors included initiation of prenatal care in the first trimester, initiation of breastfeeding at the hospital, any alcohol use in the 3 months before and during pregnancy, and any smoking in the 3 months before and during pregnancy. Women's health outcomes included gestational diabetes, depression before pregnancy, and separately, depression after pregnancy. Infant health outcomes considered were birth weight (g), preterm birth (<37 weeks of gestation), low birth weight (LBW; <2500 g), small for gestational age (SGA; <10th percentile for gestational age and sex), and large for gestational age (LGA; >90th percentile for gestational age and sex).^18^ Infant health outcomes were determined from birth weight and gestational age recorded on the birth certificate.

### Covariates

In addition to the outcome measures, a number of covariates were compared across socially assigned race groups, including women's race/ethnicity (white, black or African American, Hispanic or Latina, Asian or Pacific Islander, American Indian, other), age (≤29, ≥30), marital status (married not married), education level (high school or less, some college or more), nativity (born in the United States, born outside the United States), receipt of Special Supplemental Nutrition Program for Women, Infants, and Children (WIC) assistance during pregnancy (yes, no), sex of infant (male, female), plurality (singleton, multiple), parity (first child, second+ child), and gestational age (weeks).

Racial lived experience covariates consisted of two variables. First, how often the woman thought about her race (constantly or once a day or once a week, once a month or once a year, never). Second, whether or not the women had felt background stress, were emotionally upset, or had physical symptoms due to treatment of their race; endorsement of any of these items was considered yes versus no.

### Analysis

Women were included in the analysis whose infants were born at 30 to 44 weeks of gestation with complete information on socially assigned race and had birth weights consistent with gestational age based on the method by Alexander et al.^19^ Outcomes and covariates were compared for women with a nonwhite versus a white social ascription using chi-squared statistics. Logistic regression models were run for each of the outcomes of interest and adjusted for covariates that were significant in chi-squared comparisons: women's age, marital status, education level, WIC status, and plurality. Models for LBW, SGA, and LGA were additionally adjusted for infant sex and gestational age. Models for preterm birth were adjusted for infant sex only.

Three additional models for each outcome were conducted and adjusted for, independently, how often a woman thought about her race, experiencing symptoms due to race, and nativity status. A linear regression model was run for the continuous birth weight outcomes (in grams) and was adjusted for covariates that were significant in chi-squared comparisons: women's age, marital status, education level, WIC status, infant's sex, plurality, and gestational age.

All analyses were conducted using SAS software version 9.4 (SAS Institute, Inc., Cary, NC) without sample weights. The Boston College Institutional Review Board reviewed the study and considered it exempt.

## Results

### Sample description

A total of 5728 women who had given birth in Massachusetts between 2012 and 2015 had information on socially assigned race. Of these, 3851 (67.3%) were socially assigned as nonwhite and 1877 (32.7%) were socially assigned as white. In this sample (where women could choose more than one self-identified race), 2029 (35.4%) self-identified as white, 1124 (19.6%) self-identified as African American or black, 1404 (24.5%) self-identified as Hispanic, 1303 (22.8%) self-identified as Asian, and 363 (6.3%) self-identified as other.

### Bivariate comparisons

Compared with women socially assigned as white, more women socially assigned as nonwhite were <29 years of age (46.7% vs. 33.6%, *p*<0.05), had a high school education or less (33.7% vs. 15.8%, *p*<0.05), and relied on WIC during pregnancy (53.0% vs. 20.6%, *p*<0.05). Fewer women socially assigned as nonwhite were married (58.5% vs. 77.0%, *p*<0.05) or born in the United States (31.6% vs. 85.8%, *p*<0.05) than those socially assigned as white.

Women socially assigned as nonwhite thought about race constantly or once a day or once a week (26.1%) and experienced stress or symptoms due to their treatment by race (14.6%) more than women socially assigned as white (9.4% and 3.0%, respectively) (Table 1). Compared with women socially assigned as white, more women socially assigned as nonwhite breastfed ever (88.7% vs. 86.5%, *p*<0.05), had infants that were born preterm (10.1% vs. 7.9%, *p*<0.05), had infants born with LBW (8.8% vs. 5.7%, *p*<0.05), and infants born SGA (11.6% vs. 8.0%, *p*<0.05) (Table 1). When examining outcomes stratified by more granular socially assigned race data, we see disparities between groups on all women's health behaviors, health outcomes, and infant health outcomes (Supplementary Table S1).

**Table 1.** Study Variables Stratified by Socially Assigned Race for Mothers Giving Birth in Massachusetts Between 2012 and 2015 Who Participated in the Pregnancy Risk Assessment Monitoring System Survey

	White social ascription (***N***=1877)	Nonwhite social ascription (***N***=3851)	***p***
Sociodemographics			
Maternal age			<0.01
<29	630 (33.6)	1800 (46.7)	
30+	1247 (66.4)	2051 (53.3)	
Maternal self-identified race/ethnicity (not exclusive)			<0.01
White	1781 (94.9)	248 (6.4)	
African American/black	14 (0.8)	1110 (28.8)	
Hispanic	124 (6.6)	1280 (33.3)	
Asian/PI	45 (2.4)	1258 (32.7)	
American Indian	20 (1.1)	62 (1.6)	
Other	48 (2.6)	315 (3.2)	
Education			
High school or less	296 (15.8)	1296 (33.7)	<0.01
Singleton	1839 (98.2)	3783 (98.2)	0.74
First born	826 (44.0)	1673 (43.4)	0.94
WIC use during pregnancy	387 (20.6)	2042 (53.0)	<0.01
Marital status			
Married	1446 (77.0)	2254 (58.5)	<0.01
Mother's nativity			<0.01
Born in the United States	1611 (85.8)	1217 (31.6)	
Born outside the United States	266 (14.2)	2634 (68.4)	
Race variables			
How often do you think about your race?			<0.01
Constantly/once a day/once a week	176 (9.4)	982 (26.1)	
Once a month/once a year	373 (20.0)	809 (21.5)	
Never	1319 (70.6)	1974 (52.4)	
Felt background stress OR emotionally upset OR physical symptoms due to treatment by race?			<0.01
Yes	56 (3.0)	561 (14.6)	
No	1821 (97.0)	3290 (85.4)	
Outcomes			
Women's health behaviors			
Prenatal care in first trimester	1692 (90.1)	3373 (87.6)	<0.01
Breastfed ever	1623 (86.5)	3415 (88.7)	0.02
Alcohol consumption	755 (40.2)	496 (12.9)	<0.01
Smoking	178 (9.5)	214 (5.6)	<0.01
Women's health outcomes			
Gestational diabetes	74 (3.9)	267 (6.9)	<0.01
Depression before pregnancy	239 (12.7)	350 (9.1)	<0.01
Depression postpregnancy	93 (4.4)	251 (6.5)	<0.01
Infant health outcomes			
Birth weight (g)	3416.6 (603.4)	3230.8 (623.7)	
Preterm birth	148 (7.9)	389 (10.1)	<0.01
Low birth weight	107 (5.7)	337 (8.8)	<0.01
SGA	151 (8.0)	448 (11.6)	<0.01
LGA	180 (9.6)	214 (5.6)	<0.01

LGA, large for gestational age; PI, Pacific Islander; SGA, small for gestational age; WIC, Special Supplemental Nutrition Program for Women, Infants, and Children.

### Models

When compared with women socially assigned as white, women socially assigned as nonwhite had higher odds of breastfeeding (adjusted odds ratio [AOR]: 1.86, 95% confidence interval [CI]: 1.54 to 2.25), lower odds of consuming alcohol (AOR: 0.27, 95% CI: 0.24 to 0.31), and lower odds of smoking (AOR: 0.30, 95% CI: 0.24 to 0.38) (Table 2). Women socially assigned as nonwhite had higher odds of reporting gestational diabetes (AOR: 1.97, 95% CI: 1.49 to 2.61), but lower odds of depression before pregnancy (AOR: 0.53, 95% CI: 0.44 to 0.64) than those socially assigned as white.

**Table 2.** Logistic Regression Models Comparing Women Socially Assigned as Nonwhite with Women Socially Assigned as White for Women's Health Behaviors, Women's Health Outcomes, and Infant Health Outcomes in Massachusetts Between 2012 and 2015 Who Participated in the Pregnancy Risk Assessment Monitoring System Survey

Outcome	Model 1: Adjusted	Model 2: Model 1+thinking about race	Model 3: Model 1+symptoms due to race	Model 4: Model 1+nativity
Women's health behaviors^a^				
Prenatal care in first trimester	0.88 (0.72 to 1.07)	0.85 (0.70 to 1.05)	0.91 (0.74 to 1.11)	1.02 (0.81 to 1.27)
Breastfed ever	**1.86 (1.54 to 2.25)**	**1.74 (1.43 to 2.11)**	**1.84 (1.52 to 2.23)**	**1.31 (1.06 to 1.62)**
Alcohol consumption	**0.27 (0.24 to 0.31)**	**0.25 (0.21 to 0.28)**	**0.28 (0.24 to 0.32)**	**0.49 (0.42 to 0.58)**
Smoking	**0.30 (0.24 to 0.38)**	**0.31 (0.24 to 0.40)**	**0.30 (0.24 to 0.38)**	**0.52 (0.40 to 0.67)**
Women's health outcomes^a^				
Gestational diabetes	**1.97 (1.49 to 2.61)**	**2.08 (1.55 to 2.78)**	**1.93 (1.44 to 2.56)**	**1.69 (1.22 to 2.34)**
Depression before pregnancy	**0.53 (0.44 to 0.64)**	**0.51 (0.42 to 0.62)**	**0.48 (0.40 to 0.59)**	**0.77 (0.62 to 0.96)**
Depression postpregnancy	1.13 (0.86 to 1.49)	1.06 (0.80 to 1.41)	0.97 (0.73 to 1.28)	**1.46 (1.08 to 1.98)**
Infant health outcomes				
Birth weight (g)^b^	**−178.6 (−213.6, -143.6)**	**−186.4 (−222.5, −150.2)**	**−175.2 (210.7, −139.7)**	**−188.2 (−228.9, −147.6)**
Preterm birth^c^	1.16 (0.94 to 1.44)	1.19 (0.95 to 1.49)	1.15 (0.92 to 1.43)	1.23 (0.97 to 1.58)
Low birth weight^d^	**1.66 (1.29 to 2.14)**	**1.74 (1.34 to 2.26)**	**1.66 (1.29 to 2.15)**	**1.81 (1.36 to 2.41)**
SGA^d^	**1.46 (1.19 to 1.80)**	**1.49 (1.20 to 1.84)**	**1.44 (1.17 to 1.77)**	**1.43 (1.13 to 1.81)**
LGA^d^	**0.59 (0.47 to 0.74)**	**0.60 (0.48 to 0.76)**	**0.60 (0.47 to 0.75)**	**0.63 (0.48 to 0.82)**

Bolded estimates indicate significance at the *p*≤0.05 level.

^a^ Adjusted for age, education, WIC status, marital status, plurality.

^b^ Linear regression.

^c^ Adjusted for age, education, WIC status, marital status, plurality, sex of infant.

^d^ Adjusted for age, education, WIC status, marital status, plurality, sex of infant, gestational age.

Infant health outcomes also differed between infants born to women socially assigned white and nonwhite. In particular, mothers socially assigned as nonwhite had higher odds of giving birth to an infant born LBW (AOR: 1.66, 95% CI: 1.29 to 2.14), and/or SGA (AOR: 1.46, 95% CI: 1.19 to 1.80) when compared with women socially assigned as white. Women socially assigned as nonwhite gave birth to infants who weighed less (−178.6 g, 95% CI: −213.6 to −143.6) and had a lower odds of giving birth to an infant born LGA (AOR: 0.59, 95% CI: 0.47 to 0.74) than those socially assigned white.

After adjusting for how often a woman thought about her race, disparities persisted between nonwhite socially assigned women and white socially assigned women in ever breastfed, alcohol consumption, smoking, gestational diabetes, depression before pregnancy, and all infant health outcomes (Table 2). After adjusting for stress due to race, disparities persisted between nonwhite socially assigned women and white socially assigned women for all variables. After adjusting for nativity status, disparities persisted between nonwhite and white socially assigned women in alcohol consumption, gestational diabetes, and all infant health outcomes.

## Discussion

In a diverse sample of women giving birth in Massachusetts, we found that socially assigned nonwhite women had higher odds of ever breastfed and lower odds of smoking or consuming alcohol than women socially assigned as white. However, socially assigned nonwhite women had higher odds of gestational diabetes than women socially assigned as white. In addition, nonwhite socially assigned women had higher odds of delivering an infant that was LBW and SGA than women socially assigned white even after adjusting for how often a woman thought about her race and, independently, nativity status. In summary, women socially ascribed as nonwhite had worse health outcomes for themselves and their infants despite practicing more beneficial health behaviors.

As other authors have noted,^16^ when the differences observed in our sample between the health outcomes of white and nonwhite mothers and their infants are viewed in light of differences in opportunities and experiences due to race (i.e., racism), our results add to existing evidence that racism has a profound impact on health.^20^ In particular, our results corroborate prior work suggesting that racism, and not individual-level determinants (e.g., behaviors), is a major driver of maternal and infant health outcomes for racialized mothers and infants.

Research in settings outside of maternal and infant health has found that white social ascription has health advantages, even for individuals who self-identify as nonwhite.^16^ In our study, we found that women socially assigned as nonwhite had poorer health outcomes for both themselves and their infants despite engaging in more beneficial health behaviors including ever breastfed and reduced odds of alcohol or smoking than women with a white social ascription. These results confirmed our prior hypotheses; while some outcomes were not significant, results trended in the expected direction. These patterns could point to ways in which systemic and structural factors, such as racism and discrimination, can affect maternal and infant health, even when women engage in health promoting behaviors or adhere to treatment regimens.

Mothers socially assigned as nonwhite thought about their race more frequently and felt more background stress due to their race than mothers socially assigned as white in this sample. Many other social determinants of health varied between these two groups including education level, WIC status, and marital status. While there may be other reasons why the lived experiences of these two groups may be different, interpersonal racism has been found to be an important social determinant of health in nonwhite populations.^21^ The associations between socially assigned race and health outcomes in our study are robust even after adjusting for thinking about race and stress due to racial treatment. This finding suggests that socially assigned race has an enduring impact on health outcomes independent of treatment by race.

Furthermore, after adjusting for symptoms due to racial mistreatment, the strength of the association between socially assigned race and maternal and infant health outcomes remains relatively consistent suggesting that there may be other pathways by which the experiences of socially assigned race “get under the skin.” Although treatment by race is a fundamental component of interpersonal racism, we know that structural racism (e.g., racial segregation, voter suppression policies, environmental injustice)^22^ affects health independently of interpersonal racism.^23^ The environment in which women live and work is already molded by the consequences of structural racism, influencing their health outcomes even in the presence or absence of interpersonal racism.^24^

Nativity status is a determinant of health and health disparities.^25,26^ We found that adjusting for nativity status attenuated the association between socially assigned race and ever breastfed, alcohol consumption, smoking, gestational diabetes, depression before pregnancy, SGA, and LGA. These findings may suggest that nativity status plays a role in how individuals interpret interactions with the outside world. Foreign-born mothers may attribute interactions, be it negative or positive, to their acculturation status (e.g., language, culture), whereas US-born mothers who may have a greater awareness of race and its impact may perceive interactions from a racial lens. Future research should examine the interrelationship between self-identified race, socially assigned race, nativity, and health among mothers.

The conclusions of this work need to be understood in the context of a number of limitations. First, data on socially assigned race were only collected on the Massachusetts PRAMS surveys between 2012 and 2015; this does not allow us to make more recent comparisons and contextualize the results within the current sociopolitical climate in the United States. In particular, the recent murders of black individuals by police forces and the rise of the #BlackLivesMatter movement may influence maternal and infant health outcomes.^27^ More states should consider incorporating the question on socially assigned race into their PRAMS surveys.^28^ In addition, due to the cross-sectional nature of the PRAMS data, we were not able to conduct a formal mediation analysis to understand the potential mediating role of thinking about race, treatment by race, or nativity status on the impact of social racial ascription on women's and infant's health outcomes.

Another acknowledged limitation of this work is that examining a white versus nonwhite dichotomy did not allow us to highlight the potential for nuance in the experiences of racialized mothers. We cannot examine specific examples of racism, such as anti-blackness or misogynoir, due to the lack of ample data. We cannot suggest that all mothers socially assigned as nonwhite have the same racial lived experiences; our work attempts to understand the impact of a nonwhite socially assigned race on health. Future work is needed to examine patterns within racial and ethnic groups, including those with more than one socially assigned race, with additional detailed data on racial lived experiences beyond those asked in the Reactions to Race module.

Despite these limitations, this analysis provides evidence of differential women's health behaviors, health outcomes, and infant health outcomes by socially assigned race among a diverse group of new mothers in Massachusetts. This analysis adds to the growing evidence that a diverse set of race measures should be collected when examining racism's impact on maternal and infant health. There is abundance of evidence that discrimination exposure is associated with health-compromising behaviors (e.g., poor sleep, smoking, physical inactivity).^29,30^ While those socially assigned as nonwhite in our study had better health behavior profile compared with their white counterparts, they had poorer health. Future studies should examine other health behaviors and biological processes that may help explain the link between discrimination, as measured by socially assigned race, and maternal health behaviors. Socially assigned race can provide an important context for women's experiences that influence their health and the health of their infants.

## Supplementary Material

Supplemental data

## Abbreviations Used

AOR: adjusted odds ratio
CI: confidence interval
LBW: low birth weight
LGA: large for gestational age
PRAMS: Pregnancy Risk Assessment Monitoring System
SGA: small for gestational age
WIC: Special Supplemental Nutrition Program for Women, Infants, and Children
