# Supplementary material for: Socially Assigned Race and the Health of Racialized Women and Their Infants
Source: Health Equity. 2022 Nov 18;6(1):845–51. doi: 10.1089/heq.2022.0079 (PMC9712049; doi:10.1089/heq.2022.0079)
Supplement: Supplemental data [file Suppl_TableS1.docx]

|  | **Socially-assigned Race** | | | | |
| --- | --- | --- | --- | --- | --- |
| **Outcomes** | **White** (N=1877) | **Black** (N=1081) | **Hispanic/Latina** (N=1253) | **Asian or Pacific Islander** (N=1206) | **American Indian or Other** (N=311) |
| **Women’s Health Behaviors**, N (%) |  |  |  |  |  |
| *Prenatal care in 1st trimester* | 1692 (90.1) | 899 (83.2) | 1106 (88.3) | 1099 (91.1) | 269 (86.5) |
| *Breastfed ever* | 1623 (86.5) | 951 (88.0) | 1082 (86.4) | 1110 (92.0) | 272 (87.5) |
| *Alcohol consumption* | 755 (40.2) | 139 (12.9) | 141 (11.3) | 166 (13.8) | 50 (16.1) |
| *Smoking* | 178 (9.5) | 72 (6.7) | 92 (7.3) | 24 (2.0) | 26 (8.4) |
| **Women’s Health Outcomes**, N (%) |  |  |  |  |  |
| *Gestational diabetes* | 74 (3.9) | 67 (6.1) | 63 (5.0) | 120 (10.0) | 18 (5.8) |
| *Depression prior to pregnancy* | 239 (12.7) | 109 (10.1) | 145 (11.6) | 69 (5.7) | 27 (8.7) |
| *Depression post pregnancy* | 83 (4.4) | 86 (8.0) | 90 (7.2) | 50 (4.2) | 25 (8.0) |
| **Infant Health Outcomes** |  |  |  |  |  |
| *Birthweight (grams),* Mean (SD) | 3416.6 (603.4) | 3216.8 (706.4) | 3253.8 (669.5) | 3226.5 (499.9) | 3204.2 (556.1) |
| *Preterm birth*, N (%) | 148 (7.9) | 125 (11.6) | 138 (11.0) | 92 (7.6) | 34 (10.9) |
| *Low birth weight*, N (%) | 107 (5.7) | 111 (10.3) | 117 (9.3) | 80 (6.6) | 29 (9.3) |
| *SGA*, N (%) | 151 (8.0) | 126 (11.7) | 123 (9.8) | 155 (12.9) | 44 (14.2) |
| *LGA*, N (%) | 180 (9.6) | 75 (6.9) | 78 (6.2) | 47 (3.9) | 14 (4.5) |

**Supplemental Table**. Study outcomes stratified by (more granular) socially-assigned race for mothers giving birth in Massachusetts between 2012 and 2015 who participated in the PRAMS survey.
